# Use of Complementary and Alternative Medicine in Children with Neuromuscular Disorders Followed at Penn State Health Pediatric Muscular Dystrophy Association Care Center Clinic

**DOI:** 10.1177/08830738231186490

**Published:** 2023-07-16

**Authors:** Rea Mittal, Caroline Maltese, Elizabeth Bolt, Dustin Paul, Gayatra Mainali, Sunil Naik, Sita Paudel, Erik Lehman, Ashutosh Kumar

**Affiliations:** 1 12310 Department of Pediatrics and Neurology, Penn State College of Medicine, Hershey, PA, USA; 2 Department of Pediatrics and Neurology, 12310 Penn State College of Medicine, Hershey, PA, USA; 3 Department of Public Health Sciences, 12310 Penn State College of Medicine, Hershey, PA, USA

**Keywords:** complementary and alternative medicine, integrative medicine, muscular dystrophy, Muscular Dystrophy Association, neuromuscular, pediatrics

## Abstract

The exact prevalence of complementary and alternative medicine (CAM) use is not known in pediatric patients with neuromuscular diseases followed by any of the 150 Muscular Dystrophy Association (MDA) Care Center Clinics nationwide. This study describes the prevalence and variety of CAM usage in this population, while also assessing the prevalence of caregiver disclosure of CAM use and caregiver perception of provider support for CAM. Fifty-two caregivers of pediatric patients seen at Penn State Health's Pediatric MDA Care Center Clinic completed our online survey. Overall, 19.2% of caregivers reported CAM use by their child. Less than half of caregivers reported discussing CAM use with their child's neurologist (41.5%); however, a majority of respondents reported interest in using CAM for their child in the future (52.8%). Understanding the prevalence of CAM usage and disclosure in pediatric MDA clinics may facilitate safer use of CAM in this community.

Neuromuscular disorders involve dysfunction of the motor unit, where the abnormality or lesion can involve the anterior horn cells, nerve root, plexus, peripheral nerve, neuromuscular junction, or muscle itself.^1^ In pediatrics, there are genetic and nongenetic etiologies for neuromuscular conditions. Common genetic etiologies include dystrophinopathies, spinal muscular atrophy, myotonic dystrophy, congenital myasthenic syndrome, and Charcot-Marie-Tooth disease. Common nongenetic etiologies include Guillain Barre syndrome, myasthenia gravis, and chronic inflammatory polyneuropathy.^2,3^ Neuromuscular patients require careful management with both pharmacologic and nonpharmacologic modalities. However, families often have concerns regarding potential side effects for pharmacologic treatments or have difficulty accessing proven nonpharmacologic therapies for certain conditions such as induced movement therapy, transcutaneous electric nerve stimulation, vibratory stimulation, electromyography biofeedback, and repetitive transcranial magnetic stimulation.^4^ In addition, adherence to these pharmacologic and nonpharmacologic treatments may not result in a complete resolution of symptoms. Therefore, patients and caregivers are often in search of seemingly safer and more effective options, and often turn to complementary and alternative medicine (CAM).

CAM is also known as “integrative” medicine. As per the National Center for Complementary and Alternative Medicine (NCCAM), CAM is defined as a group of diverse medical and health care systems, practices and products that are not presently considered to be the part of conventional Western medicine (NIH 2006).^5^ The use of CAM has been very prevalent in the Eastern world and is increasing in Western populations.^6^ Results of the 2012 National Health Interview Survey (NHIS) revealed that the prevalence of complementary and alternative therapies use in children remained approximately 12%, and that nonvitamin, nonmineral dietary supplements (eg, herbal medicines, probiotics), osteopathic or chiropractic manipulation, and yoga, tai chi, or qigong were the CAM therapies used most frequently by children.^6^

However, the percentage of patients who use CAM varies geographically, across pediatric subspecialities, and between different disease pathologies. In a study that looked at CAM usage in pediatric neurology patients, CAM was perceived to be helpful in 71% of patients with epilepsy, 45% of patients with headaches, 67% of patients with a brain injury, and 20% of patients with neuromuscular diseases.^7^ Among pediatric neurology subspecialties, a study conducted at Penn State Hershey Medical Center in children with Tourette syndrome found that 69.1% of 110 patients with Tourette syndrome reported using at least 1 CAM therapy.^8^ In comparison, 27% of 313 families with children who have cerebral palsy in Canada reported CAM use.^9^ Studies focused on Duchenne or Becker muscular dystrophy pediatric patients reported CAM usage from 75% to 80% in more than 550 patients surveyed.^10,11^ These studies examined CAM uses specifically in Duchenne or Becker muscular dystrophy patients, and not in other neuromuscular disorders patients followed at MDA Care Center clinics.^10,11^

The exact prevalence of CAM use is not known in pediatric patients with neuromuscular diseases followed by any of the 150 Muscular Dystrophy Association (MDA) Care Center Clinics nationwide. This study aims to determine the prevalence of use of CAM therapies in children with neuromuscular disorders followed at our institution's Pediatric MDA Care Center Clinic. Furthermore, we aim to identify commonly used CAM therapies for these children, ascertain the frequency of caregiver disclosure of CAM usage to their provider, and understand caregiver perception of provider support for CAM. Understanding these statistics may facilitate more effective use of CAM in this community.

## Methods

Our questionnaire targeting caregivers of children with pediatric neuromuscular disorders was created after reviewing available literature from related studies. The questionnaire inquired about demographic information, neuromuscular condition, disease characteristics, treatment modalities used, CAM use, perception of CAM, as well as patient-physician discussions regarding CAM. CAM was explicitly defined in the survey as “a category of medicine that includes a variety of treatment approaches that could be classified as nonconventional medicine. These include treatments using diet therapies, herbal medicine, mind-body medicine (meditation/yoga), and body manipulation (massages/acupuncture).” This definition was provided in order to address different levels of health literacy and understanding. As the MDA Care Center Clinic treats a variety of neuromuscular conditions, conditions were grouped for more effective statistical analysis.

Pediatric patients (18 years old and younger) seen at the Penn State Health Pediatric MDA Care Center Clinic were identified through the database maintained by the MDA Care Center pediatric neurologist. From June to July 2022, we identified and attempted to contact 110 participants over the phone. Participants were emailed with consent information, where 52 parents and caregivers completed our survey. The rest of the potential participants either did not pick up the phone after 5 attempts to contact them, did not qualify for our study, or declined to participate. We removed patients who had passed away at the time of data collection from the potential participants list. We did not leave a voice message if a potential participant did not answer our call. As the phone call and survey were conducted in English, non-English speakers were excluded from this study.

Study data was collected and managed using REDCap electronic data capture tools hosted at Penn State Health Milton S. Hershey Medical Center and Penn State College of Medicine. REDCap (Research Electronic Data Capture) is a secure, web-based application designed to support data capture for research studies, providing an intuitive interface for validated data entry, audit trails for tracking data manipulation and export procedures, automated export procedures for seamless data downloads to common statistical packages, and procedures for importing data from external sources.^12^

Chi-square tests were used to look for significant associations between variables, and results were considered significant if *P* was  <.05. All analyses were performed using SAS, version 9.4 (SAS Institute, Cary, NC).

## Results

### Demographics

We had 52 participants who identified as caregivers of children with neuromuscular disorders managed by Penn State Health's Pediatric MDA Care Center Clinic completed our survey. Of the identified children with neuromuscular illnesses, 9 (17.7%) were 1-4 years old, 11 (21.6%) were 5-9 years old, 11 (21.6%) were 10-14 years old, and 20 (39.2%) were 14-18 years old. The gender distribution of children with neuromuscular illnesses was 71.7% male and 28.3% female.

The demographics of the caregivers are depicted in Table 1. More than 79% of caregivers identified as Caucasian, and 66% reported to be married. More than half of respondents reported a household income under $100,000.

**Table 1.** Patient and Caregiver Demographics.

Variable	n (%)
Patient age, y	
1-4	9 (17.7)
5-9	11 (21.6)
10-14	11 (21.6)
14-18	20 (39.2)
Patient gender	
Male	38 (71.7)
Female	15 (28.3)
Caregiver age, y	
18-30	5 (9.43)
31-40	23 (43.4)
41-50	17 (32.1)
>50	8 (15.1)
Caregiver ethnicity^a^	
African American	3 (5.66)
Caucasian	42 (79.25)
Hispanic	5 (9.43)
Asian American	3 (5.66)
Native American	1 (1.9)
Caregiver marital status	
Single	12 (22.6)
Separated/divorced	6 (11.3)
Married	35 (66.0)
Caregiver highest level of education	
Did not complete high school	2 (3.8)
Completed high school of GED equivalent	14 (26.4)
Some college education	10 (18.9)
Completed associate’s or bachelor’s degree	21 (39.6)
Completed graduate degree	6 (11.3)
Total household income	
<50k	18 (34.6)
50k-100k	16 (30.1)
>100k	13 (25)
Declined to answer	5 (9.6)

^a^ Respondents were asked to select all that apply.

### Neuromuscular Disease Characteristics and Treatment

More than 35% of the caregivers of children with neuromuscular disorders reported a diagnosis of Duchenne muscular dystrophy in this study. Table 2 demonstrates the neuromuscular disease characteristics of the child affected, such as years since diagnosis, ambulatory status, wheelchair use, noninvasive positive pressure ventilation device use, and steroid use. Participants were able to skip questions as desired; therefore, question statistics may not always total 52 participants.

**Table 2.** Neuromuscular Disease Characteristics and Treatment in our Patient Population.

Variable	n (%)	n (%) reporting CAM use
Diagnosis		
Duchenne muscular dystrophy	19 (36.5)	4 (21.1)
Non-Duchenne muscular dystrophy	8 (15.4)	0 (0)
Spinal muscular atrophy	7 (13.5)	2 (28.6)
Charcot-Marie-Tooth	7 (13.5)	2 (28.6)
Myopathy	5 (9.6)	1 (20.0)
Other	6 (11.5)	1 (16.7)
Years since diagnosis		
0-4	17 (33.3)	3 (17.7)
5-9	13 (25.9)	3 (23.1)
10-14	15 (29.4)	3 (20.0)
15+	6 (11.8)	1 (16.7)
Ambulatory status		
Walks >200 m	20 (39.2)	4 (20.0)
Walks ≤200 m or nonambulatory	31 (60.8)	6 (19.4)
Wheelchair use		
Yes	19 (82.6)	3 (15.8)
No	4 (17.4)	1 (25.0)
Noninvasive positive pressure ventilation device use		
Yes	12 (23.5)	5 (41.7)
No	39 (76.5)	5 (12.8)
Steroid use		
Yes	15 (30.2)	4 (26.7)
No	37 (69.8)	6 (16.2)

As the MDA Care Center Clinic treats a variety of neuromuscular conditions, conditions were grouped for more effective statistical analysis. Groups included Duchenne muscular dystrophy, spinal muscular atrophy, and Charcot Marie Tooth. The non–Duchenne muscular dystrophy group encompassed Becker muscular dystrophy, congenital muscular dystrophy, facioscapulohumeral muscular dystrophy, Emery-Dreifuss muscular dystrophy, limb-girdle muscular dystrophy, and congenital myotonic dystrophy. The myopathy group included congenital myopathies and Laing distal myopathy. The other group included myasthenia gravis, congenital myasthenic syndrome, Pompe disease type 2, and nonspecified.

### Prevalence and Rationale for CAM Use

Overall, 19.2% of respondents indicated past or current CAM use. Table 3 depicts the distribution of CAM therapies that users engaged in. Of the respondents, 20.8% reported using manipulative-based or body-based methods (massage, chiropractic care, osteopathic manipulation), and 20.9% reported using mind-body methods (meditation, yoga, prayer/spirituality, hippotherapy, and horse riding).

**Table 3.** CAM Therapy Used in Our Study Population.

Type of CAM therapy used	n (%)
Manipulative-based or body-based methods	
Massage	5 (9.4)
Chiropractic care	4 (7.6)
Osteopathic manipulation	2 (3.8)
Acupuncture	0 (0)
Mind-body methods	
Meditation	4 (7.6)
Yoga	3 (5.7)
Prayer/spirituality	1 (1.9)
Hippotherapy	1 (1.9)
Horse riding	2 (3.8)
Biologically based methods	
Vitamin D	1 (1.9)
Coenzyme Q10	1 (1.9)
Herbal medicine	4 (7.6)
CBD oil	3 (5.6)
Homeopathy	0 (0)
Calcium	0 (0)
Fish oil	0 (0)
Creatine monohydrate	0 (0)
Arginine	0 (0)
Medical marijuana	0 (0)
Specialized diet	
Amino acid formula	1 (1.9)
Anti-inflammatory	1 (1.9)
Gluten-free	1 (1.9)
Low sodium	1 (1.9)

Abbreviation: CAM, complementary and alternative medicine; CBD, cannabidiol.

Respondents learned about CAM in various ways as depicted in Table 4, including through their pediatrician, family, friends, internet/TV, or literature/books. Respondents chose to initiate CAM for a variety of reasons as depicted in Table 5, including dissatisfaction with their current drug treatment outcomes or side effects, cost of drug treatments, or because they wanted to try something new. For analysis purposes, the participants were provided with a list of possible reasons why they initiated CAM and were asked to select all that applied to their situation. The majority of families that reported CAM usage have used CAM for more than a year, whereas 10% used CAM for at least 1 month to a year.

**Table 4.** Modalities in Which Respondents Learned About CAM Therapies.

Variable^a^	n (%)
Pediatrician	1 (1.9)
Family	4 (7.6)
Friends	4 (7.6)
Internet/TV	2 (3.8)
Literature	3 (5.7)

Abbreviation: CAM, complementary and alternative medicine.

^a^ Respondents were asked to select all that apply.

**Table 5.** Reasons for Using CAM.

Variable^a^	n (%)
Unhappy with current drug treatment outcomes	2 (3.8)
Unhappy with current drug treatment side effects	4 (7.6)
Cost of drug treatments	2 (3.8)
Wanted to try something new	6 (11.5)

Abbreviation: CAM, complementary and alternative medicine.

^a^ Respondents were asked to select all that apply.

Certain neuromuscular disease characteristics were associated with an increased likelihood of using complementary and alternative medicine. Of children necessitating a noninvasive positive pressure ventilation disease, 5 of 12 (41.7%) reported having used CAM. Of children who did not need a noninvasive positive pressure ventilation disease, only 5 of 39 (12.8%) reported having used CAM (*P* = .043).

### Provider Patient Communication Regarding CAM

As depicted in Table 6, the majority of caregivers reported having never discussed CAM use with their child's neurologist (58.5%). Furthermore, 75.5% of respondents reported that they were not sure if their child's neurologist would support CAM for their child.

**Table 6.** CAM Perception and Future Use in Our Study Population.

CAM Interest Category	Yes, n (%)	No, n (%)	Not sure, n (%)
Are you interested in using CAM for your child in the future?	28 (52.8)	7 (13.2)	18 (34.0)
Have you discussed using CAM with your child’s neurologist?	6 (11.3)	31 (58.5)	16 (30.2)
Do you think your child’s neurologist will support CAM for your child?	12 (22.6)	1 (1.9)	40 (75.5)

Abbreviation: CAM, complementary and alternative medicine.

Of caregivers under the age of 40 years, 3.6% of them discussed CAM use with their child's neurologist. In comparison, 20.0% of caregivers above the age of 40 years discussed CAM use with their child's neurologist. All 6 caregivers who reported discussing CAM use with their child's neurologists identified as Caucasian. In addition, 66.7% of caregivers who discussed CAM use with their child's neurologist perceived that their neurologist supported CAM use for their child, vs the 16.1% caregivers who did not discuss CAM use with their neurologist yet still perceived that their neurologist supported CAM use for their child (*P* = .031).

### Caregiver Interest in Future CAM Use

More than half of respondents reporting that they would be interested in using CAM for their child in the future (52.8%) whereas 34.0% of respondents were unsure. Of the 28 caregivers interested in using CAM for their child in the future, only 11 (39.3%) caregivers believed that their child's neurologist would support CAM use for their child vs the 17 (60.7%) caregivers who did not believe that their child's neurologist would support CAM use. This compares to the 4% (n = 1) of caregivers who believe that their child's neurologist would support use of CAM out of the caregivers who were unsure about or denied interest in future CAM use for their child (*P* = .002).

## Discussion

Prevalence of CAM use by children with neuromuscular disorders followed by Penn State Health's Pediatric MDA Care Center Clinic was reported to be 19.2%. The exact prevalence of CAM use has not been studied previously in pediatric patients with neuromuscular diseases followed by any of the 150 MDA Care Center Clinics nationwide. However, previous studies targeting Duchenne or Becker muscular dystrophy populations reported higher CAM usage from 75% to 80%.^10,11^ In comparison, of the 27 patients with Duchenne or non-Duchenne muscular dystrophy in this study, only 4 (14.8%) families reported CAM use. This large difference in CAM usage prevalence among Duchenne and non-Duchenne muscular dystrophy could be due to geographical differences and the wider variety of muscular dystrophies managed by the MDA Care Center Clinics. A recent Central Pennsylvanian study cited a similar CAM usage as 12.7% within pediatric epilepsy clinics.^13^ In comparison, 41.6% of patients recognized that they were using 1 or more types of CAM in a study conducted in an outpatient pediatric neurology clinic in Rochester, Minnesota, suggesting geographical influences on CAM use.^14^

Although there was no statistical association with certain neuromuscular diseases using CAM more than others, particular disease characteristics such as needing noninvasive positive pressure ventilation were associated with increased likelihood of CAM use. This is consistent with other studies where more severe or early onset of symptoms were associated with a higher likelihood of using CAM therapy.^10^

The most commonly reported CAM therapies used included massage, chiropractic care, meditation, and herbal medicine. Massage was one of the most popular CAM therapies reported in other studies targeting pediatric muscular dystrophy patients.^10^ In a study by Nozaki et al,^15^ a jaw range of motion exercise enhanced by a hot pack placed on the masseter muscle as well as massage of the masseter improved greatest occlusal force in Duchenne muscular dystrophy patients aged 16-29 years, allowing these patients greater enjoyment of their meals. Although there has not been extensive research studying effects of CAM on pediatric neuromuscular conditions, multiple types of CAM therapies have been reported to be effective in reducing pain in adults with neuromuscular conditions such as painful peripheral neuropathy. Alleviation of pain was evident in individuals with painful peripheral neuropathy after adding dietary supplements such as B_12_, alpha lipoic acid, acetyl-l-carnitine, and vitamin D in deficient patients as well as after initiation of mind-body techniques such as acupuncture and yoga.^16^ Therefore, further research regarding efficacy of popular CAM therapies would be useful in directing care for individuals with pediatric neuromuscular disorders.

Although there are numerous possible benefits associated with CAM therapies, there are also a multitude of potential adverse effects that must be considered before the initiation of these therapies. Herbal medicines, which were popular among CAM users in this study, can have negative consequences because of various drug interactions, cancerogenic properties, and the potential for organ toxicity.^17^ Therapies such as alternative diets can be harmful in that children may be predisposed to nutritional deficiencies that lead to impairments in growth and development as well as dermatologic complications such as atopic dermatitis.^18,19^ Because of the possible unintended side effects and pharmacologic interactions of CAM therapies, caregiver disclosure to a provider is necessary for prudent management.

In our study, less than half of caregivers reported discussing CAM use with their child's neurologist (41.5%). This compares to meta-analyses citing a disclosure rate of 33% for biologically based CAM.^20^ Open communication between doctors and families regarding CAM usage should be encouraged to ensure safe and effective therapy for the patient. Therefore, it is important to understand and address reasons that caregivers may or may not disclose CAM usage to providers.

Significant associations in our study that suggested successful caregiver disclosure of CAM use with their child's neurologist included caregiver age >40 years and caregiver perception that their neurologist supported CAM use for their child. Other factors previously reported in literature to support disclosure of CAM use by patients include the perceptions that providers are knowledgeable on CAM use, that providers will be supportive regarding CAM use, and that disclosure is important for safety.^20^

There was a significant relationship between caregiver interest in using CAM in the future and perception of neurologist support of CAM use. Of the 28 caregivers interested in using CAM for their child in the future, the majority of them (60.7%) believed that their child's neurologist would not support CAM use for their child. Conversely, of the 25 caregivers unsure or not interested in future CAM use for their child, only 1 of them believed that their child's neurologist would support CAM use for their child. Caregiver perception that their child's neurologist does not support CAM use, therefore, may hinder their likelihood to discuss their interest in these therapies with their provider. Other potential reasons why caregivers may not disclose the use of or interest in CAM to their child's physician other than fear of provider disapproval include lack of inquiry by provider, perceptions that the disclosure is unimportant, and perceptions that providers are not knowledgeable regarding CAM.^20,21^ For example, only 14% of caregivers of children with autism perceived their physician to be knowledgeable about CAM in a Texas-based study.^22^ Considering that more than half of caregivers in our study reported interest in using CAM for their child in the future, it is important for providers to proactively discuss safe CAM use with families in order to create a constructive dialogue surrounding CAM benefits and concerns.

### Limitations

Limitations of this study include the convenience sample methodology, where caregivers who choose to opt in to the survey may have different experiences than those who opted out of the survey. Considering that the Penn State Health's Pediatric MDA Care Center Clinic was following around 110 patients at the time of data collection, half of which consented to this study, the sample size of our study was small, which may confound some results. Furthermore, the data from this study is specific to the prevalence of CAM use in central Pennsylvania and is not generalizable to the whole country.

There was also a selection bias for English-speaking patients. Although we attempted to collect data regarding ethnicity of the caregiver to account for demographic differences, we did not obtain information from non–English-speaking populations that means our data may not represent the full population. The Penn State MDA clinic is served by 2 child neurologists/neuromuscular specialists, where subject perceptions about their neurologists’ support for CAM use may not be generalizable to larger groups of child neurologists/neuromuscular specialists managing similar patient populations.

## Conclusion

Less than half of caregivers using CAM for their child in the Penn State Health's Pediatric MDA Care Center Clinic disclosed their CAM use to their child's neurologist. Although more than half of the caregivers surveyed are interested in using CAM for their child in the future, the majority of them believe that their child's neurologist would not support CAM use for their child. Open and proactive communication between health care providers and families regarding CAM usage, benefits, and risks should be encouraged to ensure safe and effective therapy for the patient.

## Supplemental Material

sj-docx-1-jcn-10.1177_08830738231186490 - Supplemental material for Use of Complementary and Alternative Medicine in Children with Neuromuscular Disorders Followed at Penn State Health Pediatric Muscular Dystrophy Association Care Center Clinic Click here for additional data file. Supplemental material, sj-docx-1-jcn-10.1177_08830738231186490 for Use of Complementary and Alternative Medicine in Children with Neuromuscular Disorders Followed at Penn State Health Pediatric Muscular Dystrophy Association Care Center Clinic by Rea Mittal, Caroline Maltese, Elizabeth Bolt, Dustin Paul, Gayatra Mainali, Sunil Naik, Sita Paudel, Erik Lehman and Ashutosh Kumar in Journal of Child Neurology
