# Supplementary material for: Use of Complementary and Alternative Medicine in Children with Neuromuscular Disorders Followed at Penn State Health Pediatric Muscular Dystrophy Association Care Center Clinic
Source: J Child Neurol. 2023 Jul 16;38(6-7):367–72. doi: 10.1177/08830738231186490 (PMC10466977; doi:10.1177/08830738231186490)
Supplement: sj-docx-1-jcn-10.1177_08830738231186490 - Supplemental material for Use of Complementary and Alternative Medicine in Children with Neuromuscular Disorders Followed at Penn State Health Pediatric Muscular Dystrophy Association Care Center Clinic [file sj-docx-1-jcn-10.1177_08830738231186490.docx]

1. Do you consent to this survey? Y/N
2. Is your child a: Male Female
3. How old is your child? <1, 1-4, 5-9, 10-14,14-18
4. Diagnosis:
   1. Duchenne Muscular Dystrophy
   2. Becker’s Muscular Dystrophy
   3. Spinal muscle atrophy
   4. Congenital myopathy
   5. Congenital muscular dystrophy
   6. Facioscapulohumeral muscular dystrophy
   7. Emery-dreifuss muscular dystrophy
   8. Limb-girdle muscular dystrophy
   9. Metabolic myopathy
   10. Congenital myotonic dystrophy
   11. Charcot-Marie-Tooth disease
   12. Chronic inflammatory demyelinating polyneuropathy
   13. Myasthenia gravis
   14. Congenital myasthenic syndrome
   15. Nonspecific neuromuscular disorders
   16. Other: Write in
5. Number of years since diagnosis?
6. Ambulatory status:
   1. walks > 200 m
   2. walks 50-200 m
   3. walks upto 50 m
   4. nonambulatory
7. If nonambulatory, Use wheelchair?
   1. Age first used wheelchair?
8. Noninvasive positive pressure ventilation devices use? Y/N
   1. Age first used
9. Steroid use? Y/N
10. Are you the primary caregiver? Y/N
11. How are you related to the child?
    1.  Mother Father Guardian Other……………………….
12. How old are you? 18-30, 31-40, 41-50, 50+
13. What is the highest level of education of the primary caregiver?

Did not complete high school Completed high school Some college

Completed College Graduate degree

1. What is the ethnicity of the primary caregiver:

African American Asian American Caucasian Hispanic

Native American Other: _______________

1. What is the marital status of the primary caregiver?

Single Separated/divorced Married

1. This is an optional question about total household income? Would you categorize your income as

Less than $50K $50-100K More than $100K Don’t want to answer

1. Complementary and alternative medicine, or CAM, is a category of medicine that includes a variety of treatment approaches that could be classified as non-conventional medicine. These include treatments using diet therapies, herbal medicine, mind-body medicine (meditation/yoga), and body manipulation (massages/acupuncture). Does your child currently use or have they ever used any Complementary and Alternative Medicine therapies for their neuromuscular disease? Y/N
   1. If no 🡪 skip to question 25
2. If yes, what did they use: (Check all that apply) ** Must read all
   1. Biologically based
      1. Megavitamins:
         1. vitamin D, Ca, Fish oil, Coenzyme Q10, Creatine monohydrate, Arginine, other:
         2. Currently used vs previously used
      2. Special diet 🡪
         1. Fill in
      3. Homeopathy
      4. Herbal medicine 🡪 oral or topical
         1. Fill in
   2. Manipulative and body based
      1. Massage
      2. Chiropractic care
      3. Osteopathic manipulation
      4. Acupuncture
   3. Marijuana
      1. Medical marijuana
      2. CBD oil
   4. Mind-body
      1. Meditation
      2. Yoga
      3. Prayer/spirituality
      4. Aquatherapy
      5. Hippotherapy
      6. Horse Riding
3. In your opinion, which complementary and alternative medicine modalities were the most helpful? (Write name) …………………….
4. After using complementary and alternative medicine, did you notice any change in muscle strength?
   1. Decreased Increased No change
5. How did you learn about CAM
   1. Pediatrician, family, friend, Internet/TV, Literature/Book, Other
6. Reason for use of CAM therapy?? (multiple select)
   1. List
   2. Unhappy with current drug treatment outcomes
   3. Unhappy with current drug treatment side effects
   4. Cost
   5. Try something new
7. How long did they use CAM
   1. < 1 month, 1 mo-1 yr, >1 yr
8. Has your child developed any side effects related to CAM therapies?
   1. Y, N, I don’t know
   2. 🡪 List SE for each CAM
9. If they have stopped using CAM, what is the reason for discontinuing it?
   1. Ineffective, side effects, costs, others
10. Are you interested to use complementary and alternative medicine therapies for your child in future: Y/N, I Don't know
    1. If yes, which therapies do you plan to use?
11. Have you discussed the use of CAM with your child's neurologist? Y/N/I don't know
12. Do you think your child's neurologist supports the use of CAM for your child? Y/N/I don't know
13. To ensure we do not contact you again to complete the survey, what is your email address?
